# Towards Understanding the Polymerization Process in Bitumen Bio-Fluxes

**DOI:** 10.3390/ma10091058

**Published:** 2017-09-09

**Authors:** Jan B. Król, Łukasz Niczke, Karol J. Kowalski

**Affiliations:** 1 Faculty of Civil Engineering, Warsaw University of Technology, 00-637 Warsaw, Poland; j.krol@il.pw.edu.pl; 2 General Directorate for National Roads and Motorways, 66-004 Zielona Góra, Poland; lniczke@gddkia.gov.pl

**Keywords:** bio-oil, bio-flux, oxypolymerization, reclaim asphalt pavement (RAP), warm mix asphalt (WMA)

## Abstract

Bitumen is a commonly used material for road construction. According to environmental regulations, vegetable-based materials are applied for binder modification. Fluxed road bitumen containing a bio-flux oxidation product increases the consistency over time. The efficiency of crosslinking depends on the number of double bonds and their position in the aliphatic chain of fatty acid. The main goal of this paper was to examine the structural changes taking place during hardening bitumen with bio-flux additives. Two types of road bitumens fluxed with two different oxidized methyl esters of rapeseed oil were used in this study. Various chemical and rheological tests were applied for the fluxed-bitumen at different stages of oxygen exposure. The oxidation of rapeseed oil methyl ester reduced the iodine amount by about 10%–30%. Hardening of the fluxed bitumen generally results in an increase of the resins content and a reduction of the aromatics and asphaltenes. In the temperature range of 0 °C to 40 °C, bio-flux results with a much higher increase in the phase angle than in temperatures above 40 °C in the bitumen binder. The increase in the proportion of the viscous component in the low and medium binder temperature is favorable due to the potential improvement of the fatigue resistance of the asphalt mixture with such binders.

## 1. Introduction

Bitumen is a commonly used material for construction of flexible and semi-rigid pavements. Crude oil, from which bitumen is refined, is one of the natural resources. According to the sustainability policy, it is important to replace limited raw material with the one from renewable resources, e.g., vegetable-based material. Vegetable-based materials can be applied independently, fully or partially replacing traditional materials or supporting recycling of currently used materials.

Plants are valuable source of materials that can be used to produce binders, liqueurs, or binder solvents. Among the plants that can be used for this are the trees and shrubs from which natural resins are derived or oilseeds to obtain oils.

One of the bio-oil applications can be replacement of organic origin fluxes containing volatile substances. Fluxed asphalts are used in the technology of asphalt mixtures for the production of cold-mixed asphalt and for the repair and maintenance of pavements [1], or they can be used as asphalt substitutes or rejuvenators [2,3]. The experience of using rapeseed oils and animal-origin oils shows that it is possible to apply them to pavement repairs as bitumen fluxes, but bleeding and binder drain down in the pavement can potentially occur [4]. As the aforementioned implies, it is important to select the right amount of bio-oils for the bitumen and to investigate their properties in particular with regard to the climate in which this technology is used.

Another application of vegetable additives is the rejuvenation of reclaimed asphalt pavement (RAP), which is constantly striving to increase RAP’s content while maintaining pavement durability. When using high RAP content, it is necessary to renew the properties of the RAP-contained-bitumen by using refreshing additives. As previously shown [5,6,7,8,9,10,11,12], bio-oil can successfully play the role of such rejuvenators. Vegetable oils added to aged bitumen allow the reduction of the asphaltene content in the binder, but do not change its colloidal structure. It has been found that the addition of too high an amount of vegetable oil can lead to a reduction in the thermostability of the bitumen binder and to the increase of the rutting potential of the mixture [13]. However, by optimizing the composition of binders containing bio-oils it is possible to improve the fatigue life of bitumen and asphalt mixtures, as well as to reduce the bitumen brittle fracture temperature (in single-edged notched bending (SENB) test) and thermal stress temperature (in the thermal stress restrained specimen test (TSRST)) [6,13,14]; such rheological characterization is important in bitumen modification with bioadditives, polymers, and rubber from recycling or acids [15].

Using vegetable origin additives, in particular oils, it should be remembered that most of the current applications are intended to soften or flux the bitumen. This is a convenient solution due to the good mixability of vegetable oils with bitumen, a homogeneous product with reduced stiffness is obtained. Especially in the works [4,13,16,17] there are indications of restrictions in too high a content of these fluxes due to the potential problem of pavement properties destruction in a form of a permanent deformation. The problem of too high liquefaction of the bitumen binder can be reduced by polymerizing oils in the bitumen binder. It has been found that methyl esters of fatty acids, obtained by transesterification from vegetable oils, may be used as bitumen flux for this purpose [18]. In this case, the hardening of the binder is obtained not by evaporation but by crosslinking of the flux in the presence of oxygen. 

Oxidative polymerization of vegetable oils is a free radical chain reaction, includes initiation, propagation, and termination steps [19]: Initiation RH → R• + H•; Propagation R• + O_2_ → ROO•, ROO• + RH → ROOH + R•; Termination ROO• + R• → ROOR, R• + R• → RR

Oxypolymerization leads to the crosslinking of their structural units [20,21,22,23,24]. The efficiency of crosslinking depends on the number of double bonds and their position in the aliphatic chain of fatty acid. Vegetable oils comprise a variable number of double bonds depending on the composition. The composition of the oil and, hence, its reactivity to oxypolymerization is genotype-specific and, therefore, variable and dependent on the climatic conditions [25,26].

Previous studies have shown that the liquid products obtained from oxidation of rapeseed oil methyl esters can be used as a bitumen flux [27,28]. Fluxed road bitumen containing this oxidation product increased its consistency over time, had a high ignition temperature, and displayed satisfactory adhesion to the aggregate. The limited amount of double bonds in rapeseed oil esters does not over-stiffen the asphalt mixture. It allows for a two-stage production process and full use of bio-oil potential. In the first production stage the bitumen is liquefied and refreshed, and in the second stage (service life) polymerization of the oil in the bitumen occurs. This type of two-stage technology can be used in sustainable road surfaces without compromising its performance [29,30].

The aim of the work presented in this paper was to examine the structural changes occurring during hardening bitumen with a bio-flux additive.

## 2. Materials and Methods

### 2.1. Materials

Two types of road bitumens (35/50 and 70/100 grades) produced from Uralic crude oil and vegetable origin flux were used for the study. This bio-flux was prepared in a special oxidation process. The material for oxidation was rapeseed oil methyl ester (RME) obtained by transesterification of rapeseed oil. Some of the physicochemical properties are summarized in Table 1.

Cobalt acetate (Co(CH_3_COO)_2_•4H_2_O) was applied as a catalyst of oxidation while cumene hydroperoxide (C_9_H_12_O_2_) was used as a promoter of the reaction.

### 2.2. Production Procedure

**Figure 1:**
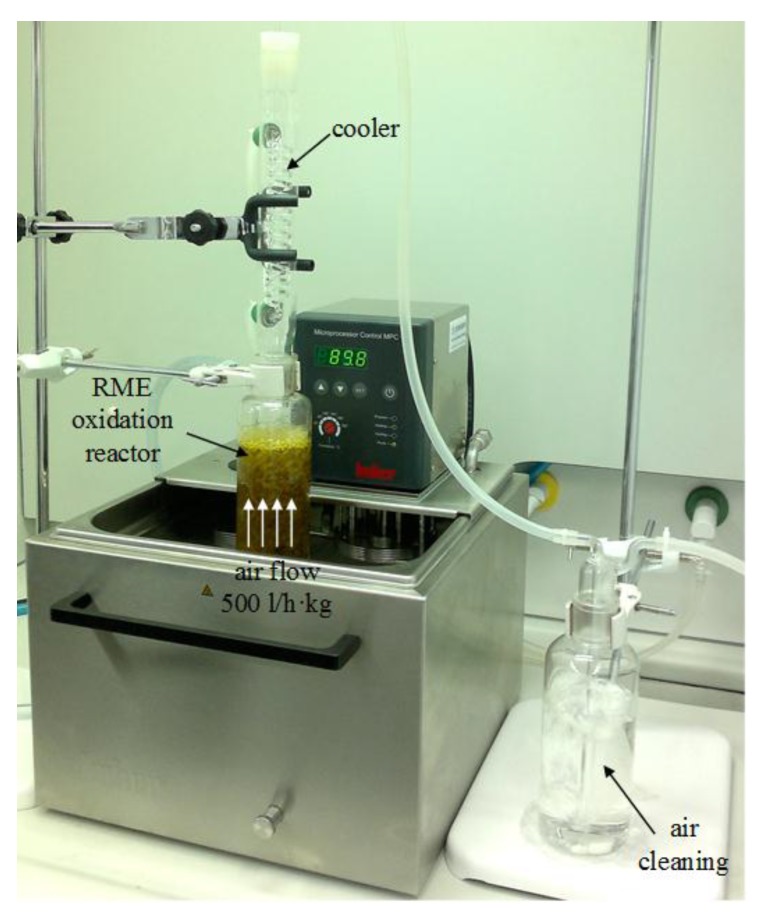
Laboratory setup for bio-agent production: reactor for oxidation of rapeseed oil esters.

Methyl esters of rapeseed oil were oxidized at 20 °C or 90 °C in a 500 mL reactor, depending on the composition (Version A or Version B, respectively). The composition and production parameters are shown in Table 2 and the reactor view is shown in Figure 1.

The glass column reactor (1000 mL) was filled with ca. 300 g of rapeseed oil methyl ester. The air inlet was at the bottom of the reactor and air was dispersed through sintered glass. The air flow rate used in the experiment (500 L kg^−1^ h^−1^) increased the air-oil phase volume by about 50%. The catalyst was added to the raw material in 0.1 wt % amounts (in terms of cobalt content) before it was subjected to oxidation. Catalytic oxidation was also performed in the presence of cumene hydroperoxide (1%/wt) used as a promoter of oxidation reactions. The liquid oxidation products were collected for analysis every 0.5 h. The volatile products were trapped in a scrubber with trichloroethylene which was placed in a dewar vessel filled with a cooling mixture.

Oxidized RME in the presence of reaction promoters was stored at 5 °C in a sealed container without light and oxygen. Modifications of bitumen were carried out by heating the binder to 150 °C in a laboratory oven. Then a bio-flux (at room temperature) was added to the hot bitumen and stirred with a glass baguette for about half a minute—methyl esters of rapeseed oil were well homogenized with the bitumen. No component separation was observed during the modification process. Test samples were taken immediately after modification, and the remaining binder was poured onto a flat tray in a thickness of approximately 1 mm for further conditioning over time. 

Samples were conditioned under room conditions. After 28 days, the binder was collected cold with a heated spatula and test specimens were formed.

### 2.3. Analysis Methods

Concentration of organic peroxide in liquid oxidation products was assessed on the basis of the peroxide value determined according to the ISO 3960 standard. The acid value was tested according to the ISO 660 standard. Iodine amount was determined according to the ISO 3961 standard.

Viscosity of the flux was measured using a Brookfield DV-II+ Pro apparatus (Brookfield Engineering Laboratories, Middleborough, MA, USA). Flash point of the flux was determined using a Marcusson flash-point apparatus (Labor Muszeripari Muvek, Esztergom, Hungary).

Road bitumen modified with bio-additive was subjected to a dynamic viscosity test at 60 °C using a Brookfield apparatus (according to EN 13302:2010) and tests with a dynamic shear rheometer MCR 101 (Anton Paar GmbH, Graz, Austria) were conducted according to AASHTO T 315 (Standard Method of Test for Determining the Rheological Properties of Asphalt Binder Using a Dynamic Shear Rheometer (DSR)). Tests in DSR apparatus were conducted in a set of two parallel 25-mm diameter plates, gap 1 mm, in a temperature range between 0 °C and 100 °C, with strain between 0.1% and 12%, depending on the test temperature.

The main test method to evaluate to chemical changes during bitumen hardening was Fourier transform infrared spectroscopy (FTIR), combined with attenuated total reflection (ATR). ALPHA FTIR device was used with the diamond ATR crystal (Bruker Corporation, Billerica, MA, USA). Infrared spectroscopy was applied to the analysis of original bitumen, as well as the bitumen with bio-flux. The fractional composition of bitumens (asphaltenes, resins, aromatics, and saturates) was determined by the thin-layer chromatography with a flame-ionization detection (TLC-FID). For the analysis an IATROSCAN MK-5 (IATRON Laboratories, INC., Tokyo, Japan) apparatus equipped with Chromarod was used.

## 3. Results

### 3.1. Physicochemical Changes

**Figure 2:**
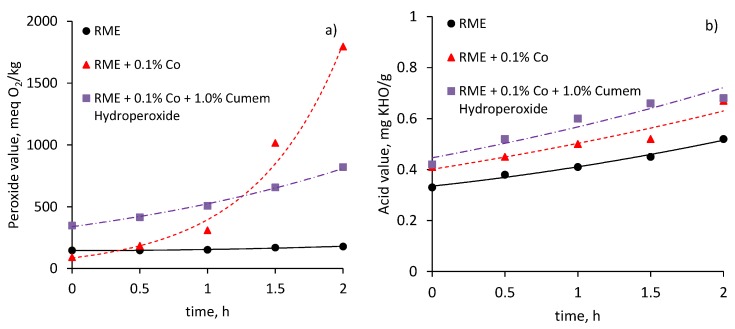
Changes in peroxide (**a**) and acid (**b**) value of rapeseed oil methyl ester vs. time of oxidation.

The changes in the peroxide value of liquid products with oxidation time are plotted in Figure 2a. The highest concentration of peroxides is observed after approximately 2 h of oxidation in the presence of a cobalt catalyst. The addition of cumene hydroperoxide into the raw material to be oxidized in the presence of a cobalt catalyst enhances the generation of peroxides at the initial stage of oxidation. The rise in their concentration observed during the first hour of oxidation is an indication of the prevalence of the rate of peroxide generation over their decomposition at this stage. The oxidation of rapeseed oil methyl esters without additives suggests a minimal increase in the peroxide value.

The changes in acid value confirm the scission of fatty acid methyl esters with the formation of carboxylic groups. The oxidation time resulted in approximately linear changes in the acid value which approached 0.7 mg KOH/g after 2 h of oxidation (Figure 2b).

Final physicochemical properties of oxidation liquid products are summarized in Table 3.

Data presented in Table 3 suggest an increase in the viscosity of liquid products. The oxidation of rapeseed oil methyl ester reduced the iodine number of the liquid products by about 10% and 30%. The addition of a promoter (hydroperoxide) results both in higher consistency and faster consumption of the double bonds in fatty acid methyl esters.

### 3.2. Structural Changes and Fractional Composition

**Figure 3:**
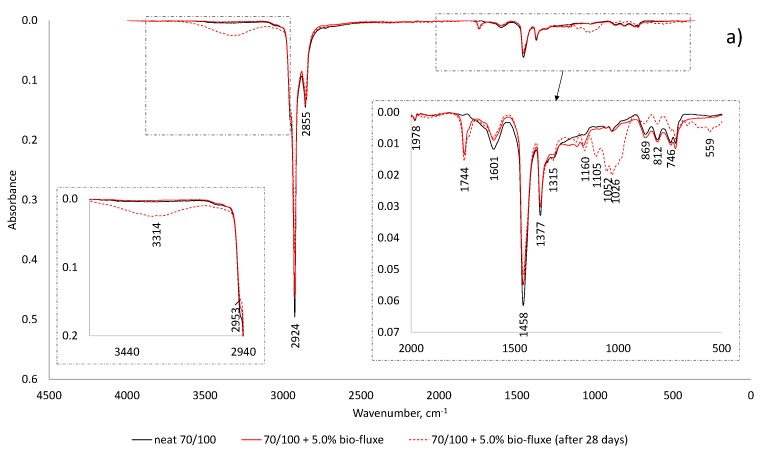

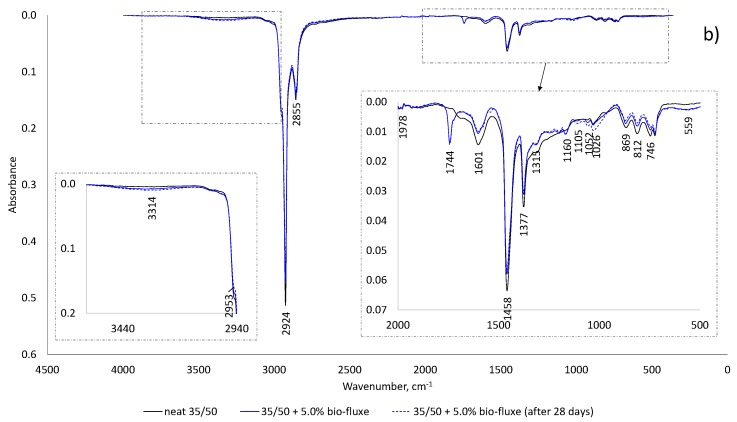
FTIR spectra of neat bitumen and fluxed bitumen by rapeseed oil methyl ester: (**a**) 70/100 bitumen; and (**b**) 35/50 bitumen.

Structural changes that have occurred upon hardening fluxed bitumen are shown in the infrared spectra of original (neat) bitumen and binder fluxed by the oxidation of the liquid products of rapeseed oil methyl esters in the presence of a cobalt catalyst (Figure 3).

The formation of oxygen-bearing compounds is evident in the FTIR spectrum of fluxed bitumen after 28 days of hardening, which exhibits a broad absorbance region at 3500–3000 cm^−1^ (νO–H) and is not observed in the original sample. Additionally, the spectrum of hardening fluxed bitumen shows a broadening of the bands centred at 1160 cm^−1^, corresponding to νC–O and δO–H vibrations of ethers and alcohols. The signal around 560 cm^−1^ is almost always assigned to the formation of C–C=O bonds in ketones and aldehydes. The visible broadening of the band around 1740 cm^−1^ in the FTIR spectrum of fluxed bitumen (Figure 3a,b), corresponds to the stretching vibrations of the carbonyl group (νC=O) in fatty acid methyl esters added to bitumen. 

The absorption band at 1600 cm^−1^ derives from the aromatic rings of bitumen compounds. The region 2960–2850 cm^−1^ is typical for CH_2_ and CH_3_ stretching of saturated compounds.

**Figure 4:**
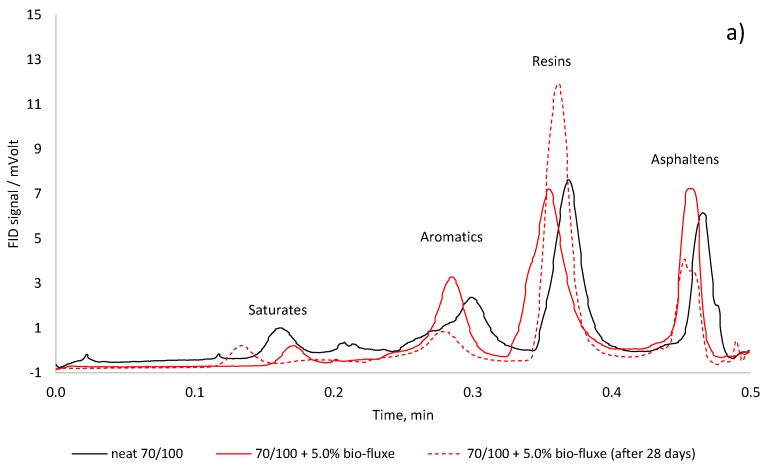

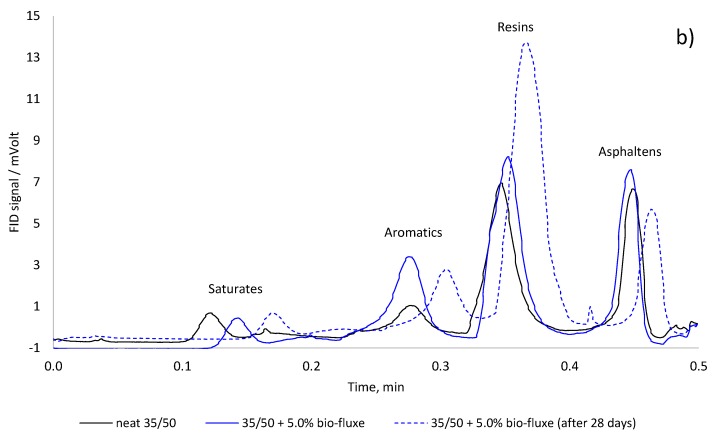
TLC-FID chromatograms of neat bitumen and fluxed bitumen by rapeseed oil methyl ester: (**a**) 70/100 bitumen; and (**b**) 35/50 bitumen.

The effect of hardening on the chemical composition of fluxed binders are present in the TLC-FID chromatograms (Figure 4).

The hardening of fluxed bitumen generally results in an increase of the resins content and a decrease of the aromatics and asphaltenes. However, the content of saturates change slightly due to their inert nature to oxygen.

### 3.3. Physical and Viscoelastic Properties of Fluxed Bitumen

**Figure 5:**
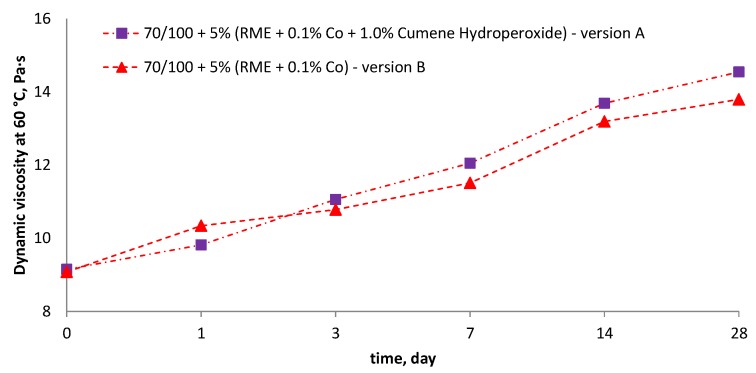
Preliminary tests of reactivity of bio-fluxes in time based on dynamic viscosity test at 60 °C.

70/100 road bitumen was modified with two bio-fluxes (Version A and B) to compare the binder hardening process over time. Figure 5 shows the results of dynamic viscosity tests at 60 °C as a function of air influence time. It can be observed that both samples increase their viscosity over time. The initial viscosity values of the two binders are not different from each other because the bio-fluxes in the initial phase (once added to the bitumen) causes the same fluxing effect. During the exposure of the binder to air there is a gradual reaction of saturating unsaturated bonds in the bio-flux and increasing the viscosity of the bitumen. Over 50% viscosity increase after 28 days was found for both the cobalt modifier, as well as the cobalt modifier with cumene hydroperoxide. Hardening of bitumen is caused by the presence of bio-flux. As shown in another publication [28], hardening of neat bitumen (in previous research evaluated based on the complex modulus G*), after 28 days was equal to only 8%. Due to the plot clarity, data for 70/100 bitumen were not shown in Figure 5, due to a high difference in the dynamic viscosity at 60 °C between neat 70/100 bitumen (191 Pa∙s) and fluxed bitumen (9 Pa∙s). The difference between the cure efficiency of both fluxes is 7%. Due to the danger of explosions at high temperature of the cumene hydroperoxide containing bio-flux, a bio-flux produced at 90 °C and containing only cobalt siccative was used for further studies (version B).

**Figure 6:**
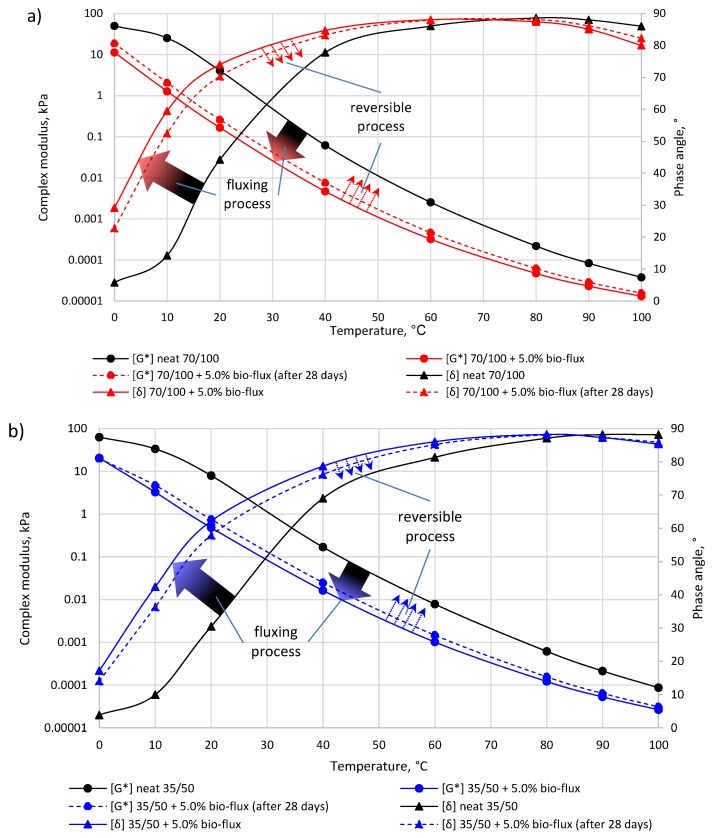
Fluxing and reversibility processes of bio-fluxed bitumen based on the dynamic shear test: (**a**) 70/100 bitumen; and (**b**) 35/50 bitumen.

The efficiency of fluxing and curing of bio-fluxed binders was investigated using a dynamic shear rheometer (DSR). The tests were performed over a wide temperature range of 0 °C to 100 °C. Figure 6 shows the results of a complex modulus (G*) and phase shift (δ) determination for two binders with different consistencies, i.e., for soft 70/100 bitumen in Figure 6a, and for harder 35/50 bitumen in Figure 6b.

Based on Figure 6 it can be stated that the bio-flux additive reduces the stiffness of the bituminous binder in a wide temperature range for both 70/100 and 35/50 binders. Binder modification with 5% addition of the bio-flux results in a decrease in the value of the complex modulus by an order of magnitude (G*) of 1 × 10^−1^ kPa over the entire range of tested temperatures. It should be emphasized that the G* change by 1 × 10^−1^ kPa corresponds approximately to one change of one bitumen grade (according to the EU bitumen classification system). Taking into account the logarithmic nature of changes in the properties of asphalt binders, this level of change should be regarded as typical.

The results of the phase shift (δ) determinations shown in Figure 6 also indicate that the addition of 5% bio-flux causes a change in its value throughout the wide temperature range. This change is different as compared to the aforementioned, however, and depends on the temperature value. In the temperature range of 0 °C to 40 °C, bio-flux produces a much higher increase in the phase angle than in temperatures above 40 °C in the bitumen binder. The increase in the proportion of the viscous component in the low and medium binder temperatures is favorable due to the potential increase of the fatigue resistance of the asphalt mixture with such binders.

## 4. Discussion

Catalytic oxidation of the rapeseed oil methyl ester results in a consistency increase, which should be attributed to the disappearance of the most reactive double bonds after two hours of oxidation. This finding is confirmed by the decrease in the iodine value. The observed fall in the content of double bonds in rapeseed oil methyl ester upon oxidation gives evidence that some part of these bonds was engaged in oligomerization and/or was oxidatively decomposed. The viscosity weight is about 1.3 and 1.8 times that of the raw material, which substantiates only a partial oligomerization of the molecules of unsaturated fatty acid methyl esters. Oligomers are formed mainly at the stage of peroxide generation. It can be assumed that peroxides are intermediate structures in the oxyoligomerization of unsaturated fatty acid methyl esters. A higher viscosity of the liquid oxidation products is attained when the reaction is carried out in the presence of cumene hyperoxide. When the flux containing this hydroperoxide is heated during flash point determination, cumene hydroperoxide decomposes (its half-life temperature is decreased by the cobalt catalyst), and the hydroxyl radicals formed initiate scission of the double bonds. This results in the formation of low molecular compounds with a flash point lower than those of the esters.

**Figure 7:**
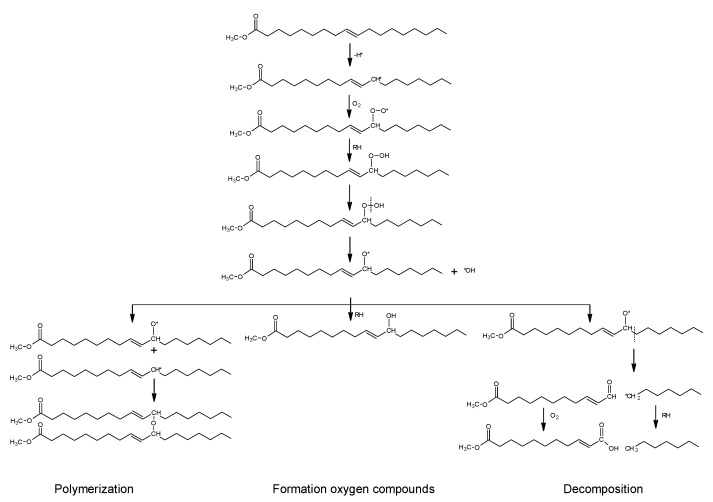
Reaction pathways of catalytic oxidation of oleic acid methyl ester.

Oxidized rapeseed oil methyl ester is a reactive flux, and hardening is possible once mixed with bitumen. After 28 days of conditioning, structural changes are confirmed by effective formation of oxygen organic compounds in fluxed binders. The absorption bands of hydroxyl, aldehydes, ketones, ether, and carboxylic group are evidenced in the FTIR spectra. The hardening of the binder after application is obtained by further polymerization of the fluxes’ vegetable-based components during consumption of molecular oxygen from the air. The reaction schemes for the formation of higher molecular and decomposition products are presented in Figure 7.

The kinetic reaction of oxidative polymerization in the bituminous medium depends on the diffusion of oxygen and it is slower in binders with higher consistency. The observed intensity bands of the oxygen functional group are more noticeable in the spectrum of fluxed 70/100 bitumen than for harder 35/50 binder.

Similar to the FTIR observation are the results of the fractional analysis of the fluxed binders. After 28 days of conditioning the increased concentration of resins suggests higher molecular polarity in fluxed binder. The changes in the fractional composition with reduction of the asphaltenes content improves the colloidal stability of the fluxed bitumens after stabilization.

Based on the rheological curve shown in Figure 6 (dashed lines), changes in the complex modulus curve (G*) and phase angle (δ) can be observed for the binders exposed to air for 28 days. Exposure to the air of the thin layer of bitumen causes the oxypolymerization reaction. It can be seen that both asphalt 70/100 and asphalt 35/50 are partially reconstructed for the original properties of the bitumen. This can be determined by analyzing the position of the binder curves after 28 days (dashed line), which runs between clean bitumen and bitumen with added bio-flux tested immediately after mixing. The shift of G* and δ curves for bio-fluxed bituminous binders towards the clean bitumen curve demonstrates overlapping polymerization in the modified bituminous binder under the influence of oxygen from the air.

## 5. Conclusions

Based on the conducted tests and analysis, the following conclusions can be drawn:

1. The most characteristic changes in the rapeseed oil methyl ester structure upon catalytic oxidation are the formation of organic peroxides and hydroperoxides (intermediate products) and the loss of the unsaturated bonds in the fatty acid methyl ester.
2. The cumene hydroperoxide in the presence of a cobalt catalyst is a more effective promoter of oxidative polymerization.
3. The pocess is time-dependent. The hardening of the bio-fluxed binders with time results in oxidative polymerization process of unsaturated fatty acid methyl ester with the formation of oxygen compounds.
4. The application of oxidized rapeseed oil methyl esters to bitumen reduces the asphaltene content and stabilizes the colloidal structure of the binder.
5. Twenty-eight day oxygen exposure to bio-fluxed bitumen results in approximately 25% recovery of the original binder stiffness.
6. The fuxing effect is particularly advantageous in the temperature range of 0 °C to 40 °C, which can potentially increase the fatigue life of the bitumen binder.

## Figures and Tables

**Table 1:** Physicochemical properties of rapeseed oil methyl ester.

Properties	Rapeseed Oil Methyl Ester
Density at 25 °C, g/cm^3^	0.8787
Flash point, °C	197
Dynamic viscosity at 25 °C, Pa∙s	0.006
Iodine number, g I_2_/100 g	116
Acid value, mg KOH/g	0.29

**Table 2:** Bio-agent types.

Compounds	Version A	Version B
Rapeseed methyl esters (RME)	98.9%	99.9%
Cobalt(II) acetate tetrahydrate catalyst	0.1%	0.1%
Cumene hydroperoxide	1.0%	-
**Process parameters**		
Oxidation temperature	20 °C	90 °C
Process duration	2 h	1 h

**Table 3:** Physicochemical properties of the oxidation of liquid products.

Properties	Liquid Product of Oxidation	
	RME + 0.1% Co + 1% Cumene Hydroperoxide (Version A)	RME + 0.1% Co (Version B)
Dynamic viscosity at 25 °C, Pa∙s	0.011	0.008
Iodine number, g I_2_/100 g	82	102
Acid value, mg KOH/g	0.68	0.67
Flash point, °C	136	191
